# Assessment of Rapid Diagnostic Tests for Typhoid Diagnosis and Assessment of Febrile Illness Outbreaks in Fiji

**DOI:** 10.4269/ajtmh.21-0771

**Published:** 2021-11-29

**Authors:** Aneley Getahun Strobel, Stephanie Airs, Cattram Nguyen, Taina Rokobuli Vadei, Silivia Matanitobua, Mike Kama, Conall H. Watson, John A. Crump, Edward Kim Mulholland, Richard A. Strugnell, Christopher M. Parry

**Affiliations:** ^1^ School of Public Health and Primary Care, College of Medicine, Nursing and Health Sciences, Fiji National University, Suva, Fiji;; ^2^ Peter Doherty Institute for Infection and Immunity, University of Melbourne, Melbourne, Australia;; ^3^ School of Tropical Medicine and Global Health, University of Nagasaki, Nagasaki, Japan;; ^4^ Murdoch Children’s Research Institute, Melbourne, Australia;; ^5^ Department of Pediatrics, University of Melbourne, Melbourne, Australia;; ^6^ Fiji Ministry of Health and Medical Services, Suva, Fiji;; ^7^ London School of Hygiene and Tropical Medicine, London, United Kingdom;; ^8^ Centre for Tropical Medicine and Global Health, University of Oxford, Oxford, United Kingdom;; ^9^ Centre for International Health, University of Otago, Dunedin, New Zealand;; ^10^ Clinical Sciences, Liverpool School of Tropical Medicine, Liverpool, United Kingdom

## Abstract

Typhoid is an endemic in Fiji with increases observed since the early 2000s and frequent outbreaks reported. We assessed the diagnostic accuracy of currently available typhoid rapid diagnostic tests (RDTs) (TUBEX, Typhidot Rapid, and Test-It assay) to establish their performance against blood culture in Fiji and to examine their suitability for rapid typhoid outbreak identification. The performance of RDTs was assessed in the public health reference laboratory in Suva, Fiji, according to the manufacturers’ instructions. A simulation was used to examine the potential use of RDTs for attribution of a febrile illness outbreak to typhoid. For the diagnostic evaluation, 179 patients were included; 49 had blood culture–confirmed typhoid, 76 had fever as a result of non-typhoid etiologies, and 54 were age-matched community controls. The median (interquartile range) age was 29 (20–46) years. Of the participants, 92 (51.4%) were male and 131 (73.2%) were indigenous Fijians. The sensitivities of the tests were 77.6% for TUBEX, 75.5% for Typhidot Rapid, and 57.1% for Test-It assay. The Test-It assay had the highest specificity of 93.4%, followed by Typhidot Rapid 85.5% and TUBEX 60.5%. Typhidot Rapid had the best performance in the simulation for attribution of a febrile illness outbreak to typhoid. Typhoid RDTs performed suboptimally for individual patient diagnosis due to low sensitivity and variable specificity. We demonstrate that RDTs could be useful in the field for rapid attribution of febrile illness outbreaks to typhoid. Typhidot Rapid had the best combination of sensitivity, specificity, positive and negative predictive values, cost, and ease of use for this purpose.

## INTRODUCTION

Typhoid is endemic in Fiji with increases observed since 2005.^1^^,^^2^ The disease is prevalent in peri-urban slums and rural villages with localized outbreaks often associated with flooding after cyclones^2^^,^^3^ or social gatherings such as funerals and weddings.^4^^,^^5^ Poor sanitation and consumption of unsafe water and unwashed produce have been identified as the risk factors for endemic typhoid.^6^^,^^7^ A serosurvey for anti-Vi-antibody suggested that the prevalence of *Salmonella typhi* exposure is much higher than that indicated by culture-confirmed cases.^8^ Acute febrile illnesses such as leptospirosis, dengue, and bacteremia because of pathogens other than *S. typhi* are also common in Fiji and may be difficult to distinguish from typhoid.^9^^,^^10^ Blood culture is the mainstay of typhoid diagnosis in Fiji, and none of the commercially available typhoid rapid diagnostic tests (RDTs) are currently in use in public health facilities. Establishing a typhoid diagnosis in patients is essential because a delay in starting effective antimicrobial treatment is a risk factor for the development of severe and complicated disease, and further transmission through prolonged shedding.^11^^,^^12^ Also, rapid diagnosis will avoid delays in the public health response to outbreaks of febrile illness, such as the application of water, sanitation, and hygiene (WASH) activities or vaccination, while the etiology is being established.

The diagnosis of typhoid is complicated by the lack of sensitivity of existing methods.^13^ Blood culture is the most common method used and its sensitivity ranges from 40% to 66%.^13^^–^^15^ The volume of blood inoculated, and prior antimicrobial therapy, influence its performance. Blood culture provides a definitive diagnosis, and yields an isolate for antimicrobial susceptibility testing, but the length of time required for blood culture to yield a result limits its value for individual patient diagnosis and for the rapid attribution of febrile illness outbreaks to typhoid. Several antibody detection RDT for typhoid are available, but their value for routine individual patient management is limited by their lack of sensitivity and specificity.^13^^,^^16^^–^^20^ Little attention has been given to the use of typhoid RDTs in determining the etiology of a febrile illness outbreak, a common public health problem in Fiji and elsewhere. In typhoid outbreaks in Fiji, the number of cases usually varies from 15 to 30 patients.^4^^,^^21^ When they occur in an area remote to a blood culture service confirming the diagnosis is a challenge. In such circumstances, an RDT with inadequate sensitivity and specificity to guide individual patient management might still be useful for rapid identification of a typhoid outbreak because of the likelihood of a common etiology for the outbreak and a larger number of persons available for testing.

We evaluated the diagnostic accuracy of available typhoid RDTs on stored serum samples from patients and subjects in Fiji. We then sought to examine if any are suitable to rule in or out typhoid as the cause of an outbreak.

## MATERIALS AND METHODS

### Study setting.

Fiji is an archipelago in the South Pacific Ocean. According to the 2017 census, the Fiji population was 884,887.^22^ Most of the population reside on the islands of Viti Levu, where the capital Suva is located, and Vanua Levu. Indigenous Fijians or i-Taukei represent 57% of the population, with Fijians of Indian Decent 37% and other ethnicities 6%.^23^

Health services are provided mainly by the Ministry of Health and Medical Services (MoHMS). There are four health divisions: Central, Western, Northern, and Eastern. Each division is further divided into subdivisions, medical areas, and then zones. There are 3 divisional hospitals and 2 specialist hospitals, 19 subdivisional hospitals, and 86 health centers.^24^

### Study subjects.

We conducted a retrospective diagnostic accuracy study using stored serum samples. Cases were patients with blood culture–confirmed typhoid diagnosed from the Central division of Fiji from March through August 2016. Controls were patients with febrile illness enrolled during the same period as cases, from the Central, Northern, and Western Divisions of Fiji, in the following four groups: those with bacteremia as a result of bacterial pathogens other than *S. typhi* confirmed by blood culture, leptospirosis confirmed by *Leptospira* IgM enzyme-linked immunosorbent assay (ELISA) (*Leptospira* IgM Standard Diagnostics, Yongin-si, South Korea), dengue confirmed by NS1 antigen or IgM ELISA (Dengue Duo, Dengue NS1 Ag+AB combo, Standard Diagnostics, Yongin-si, South Korea), and febrile patients with negative blood culture and serologic tests for leptospirosis and dengue. For community controls, we randomly selected serum samples of individuals who participated in the 2013 population-based serosurvey. Community controls reported no history of fever at the time of the survey and had not been diagnosed with typhoid in the 12 months before the survey.^8^ Samples for community controls were matched by age ± 5 years to typhoid cases in this study. The level of anti-Vi IgG antibody levels were determined for the community controls as part of the previous survey.^8^ The purpose of the community controls was to indicate the background levels of antibodies in the Fiji population as determined by the RDTs.

### Laboratory procedures.

Blood cultures and serum samples were collected from febrile patients with suspected infection as a part of routine clinical care. The blood cultures were processed using a BaCT/ALERT 3D (Biomerieux, Marcy L’Etoile, France) system at the Colonial War Memorial Hospital (CWMH), the main hospital in Fiji’s capital, Suva.

Serology testing for leptospirosis and dengue were conducted at Fiji national public health reference laboratory according to the manufacturer’s instructions. Residual serum samples of cases and controls archived at −20°C or −80°C in CWMH and public health reference laboratory were used for testing.

### Typhoid rapid diagnostic tests.

Serum samples were thawed, and an aliquot was transferred to a new tube. The samples were then de-identified according to a computer-generated randomization code and relabeled with a study number so that the laboratory staff testing the samples would be blind to the original diagnosis. The three RDTs included in this study were TUBEX^®^ test (IDL Biotech AB, Bromma, Sweden), Typhidot Rapid IgM test (Reszon Diagnostics International Sdn Bhd, Malaysia), and Test-It^TM^ Typhoid IgM test (Life Assay Diagnostics (Pty), Cape Town, South Africa). The TUBEX test is an inhibition magnetic binding immunoassay that detects *S. typhi* anti-09 IgM antibodies in patient’s serum. The semiquantitative colorimetric reading is given a score between 0 and 10 with a score ≥ 4 positive and a score of ≤ 2 negative. A score between two and four was considered indeterminate. If the serum is hemolyzed, it is not possible to determine the color. The Typhidot Rapid IgM test is a lateral flow assay that detects IgM antibodies against an outer membrane protein of *S. typhi*. A test line, in combination with a control line, is considered as a positive result. The Test-It^TM^ Typhoid IgM test is also a lateral flow assay that detects IgM antibodies against the lipopolysaccharide antigen of *S. typhi*. A test line, in combination with a control line, is considered as a positive result. The test line is scored 1+ to 4+ according to the intensity of the line.

The samples were tested in the Fiji national public health reference laboratory from September 7 to 23, 2016, using the RDTs according to the manufacturers’ instructions by laboratory personnel, trained in the use of all tests. Each test result was read independently by two separate observers to determine the inter-rater agreement. The tests were randomly relabeled and examined again by one of the observers to assess the intra-rater agreement. Information about the test characteristics, including sample volume, consumable required, storage, and cost for each test was recorded. Laboratory personnel also provided further details on ease to perform and interpret results. As the RDT’s will be used in the field, we classified the ease of use depending on the practicality of the tests, how much training would be required before use in the field, and the need for additional laboratory supplies or equipment. For visual interpretation, we assessed how recognizable are color changes or the positivity marker and any discrepancy of results between laboratory personnel. RDTs were reported as easy to use or interpret if testing procedures and results were straightforward or uncomplicated and no additional training is needed; moderate if some problems and confusion occurred during testing or interpretation; or difficult if tests required several steps to perform or complex to interpret due to indeterminate or unclear results or if extra skill or equipment are needed for testing or interpretation of results.

### Sample size.

We included samples from participants collected from March through August 2016 and determined the precision of sensitivity and specificity estimates based on the available sample size. Conservatively assuming the RDTs have a sensitivity of 60%, a sample size of 49 typhoid-positive cases would produce a sensitivity estimate with a two-sided 95% CI using the normal approximation of width ±13.7%. Similarly, assuming a specificity of 60%, 76 typhoid negative cases would produce a two-sided 95% CI of width ±11%. Higher sensitivity and specificity values would result in narrower CIs.

### Statistical analysis.

Data were entered into Microsoft Excel (Microsoft Corporation, Redmond, WA) for analysis. Sensitivity, specificity, positive predictive value (PPV), and negative predictive value (NPV) were calculated for each RDT using standard methods.^25^^,^^26^ This analysis assumed that a positive blood culture is the reference standard for typhoid diagnosis. That is, the cases with culture-confirmed typhoid and febrile controls were the reference standard positives and negatives, respectively. For the calculation of PPV and NPV, the background disease prevalence was set at 20% based on the blood culture positivity rate in health facilities.^27^ Intra-rater and inter-rater agreement for the RDT result was determined using the Kappa statistics.^25^^,^^28^ Calculated kappa values of ≤ 0.20 are considered to reflect poor agreement, > 0.20 and ≤ 0.40 fair agreement, > 0.40 and ≤ 0.60 moderate agreement, > 0.60 and ≤ 0.80 good agreement, and > 0.80 very good agreement.^25^^,^^28^^,^^29^ Intra-observer reproducibility was determined by comparing the RDT result for each observer with their RDT result on a separate reading. Inter-observer reproducibility was determined by pairing the RDT result by each observer with that of the other observer. Age and Vi IgG levels in community controls positive and negative by typhoid RDTs were compared by the Kruskal–Wallis test.

We also examined the use of binomial trials for attributing a febrile illness outbreak to typhoid.^30^ This involved using binomial distributions to estimate the probabilities of observing a certain number of typhoid-positive tests by chance alone. A simulation was used to assess the operating characteristics of the RDTs, and to examine their potential use in a typhoid outbreak. In the simulations, we created datasets of size *N* = 15 patients, which was considered to be a realistic number of patients seen and tested when doing a field visit to a village in Fiji during a suspected outbreak. We assumed that, in the absence of an outbreak, the underlying background prevalence of typhoid among febrile patients was 10%. We also assumed that an outbreak would be declared if six or more patients (i.e., ≥ 40%) tested positive by any method for typhoid. In the simulations, the number of positive tests by blood culture (*n*_pos_) were varied from 1 to 14. The true positives (TP) were drawn from a binomial distribution, $\mathrm{TP}∼\mathrm{Binomial}(n_{\mathrm{pos}},\;\mathrm{Sensitivity})$. The true negatives (TN) were drawn from a binomial distribution, $\mathrm{TN}∼\mathrm{Binomial}({15-n}_{\mathrm{pos}},\;\mathrm{Specificity}$). For the simulations, we used the sensitivity and specificity estimates observed in our study. For each scenario, 2,000 datasets were generated, and the following data were collected: the number of positives for each RDT, and whether a typhoid outbreak would have been declared based on a threshold of six positives.

This study received ethics approval from the Fiji National Health Research Ethics Review Committee (2016.63NW). The laboratory testing was performed on anonymized stored serum samples, which have been collected as part of routine clinical care. The defined clinical syndrome, age, and sex data were obtained from the laboratory forms. There was no contact with patients, so no consent was taken.

## RESULTS

Of 179 total participants, 92 (51.4%) were male and 131 (73.2%) were from i-Taukei ethnic group (Indigenous Fijians). The median (interquartile range [IQR]) age was 29 (20–46) years. The demographic profiles of cases and controls are shown in Table 1. Of the 179 participants, 49 (27.4%) had blood culture–confirmed typhoid, 76 (42.5%) had fever due to another or unknown etiology, and 54 (30.2%) were age-matched community controls. Among 76 participants with fever other than typhoid, 24 (31.6%) had dengue fever, 24 (31.6%) leptospirosis, 2 (2.6%) mixed dengue and leptospirosis infection, 14 (18.4%) bacteremia other than *S. typhi* (*Escherichia coli* (*N* = 5),* Staphylococcus aureus* (*N* = 4), β-hemolytic streptococci (*N* = 2), and one case each of *Candida* species, *Enterobacter cloacae*, and *Serratia marcescens*), and 12 (15.8%) were blood culture and serology test negative. Of the 49 typhoid cases, 24 had a leptospirosis IgM positive or dengue NS-1 or IgM positive result, suggesting possible coinfection. In the face of a positive blood culture for *S. typhi*, these may represent false positive results due to previous exposure.

**Table 1:** Demographic profile of cases and controls, Fiji, 2016

Demography	Total *N* = 179	Typhoid cases *N* = 49	Febrile controls *N* = 76	Community controls *N* = 54
Age (median [IQR])	29 (20.0–46.0)	24.5 (16.3–35.5)	37 (25.0–56.0)	26 (18.8 − 30.6)
Sex (*n*, % male)	92 (51.4)	24 (49.0)	43 (56.6)	25 (46.3)
Ethnicity (*n*, %, i-Taukei)	131 (73.2)	47 (95.5)	41 (53.9)	43 (79.6)

IQR = interquartile range.

Table 2 shows the characteristics of the three RDTs used in the study. The TUBEX test is a semiquantitative test that came with one positive and one negative control as well as detector and indicators reagents. The kit did not include consumables; therefore, additional supplies and equipment are needed to perform the test. Our laboratory staff found that the TUBEX test was technically more challenging to perform. Furthermore, the color endpoint of the TUBEX reaction was difficult to read, particularly if there was any hemolysis of the sample. This difficulty led to one in five results being indeterminate using TUBEX. The Typhidot Rapid and Test-It assay are simple lateral flow tests that were easy to perform and interpret. The intra-rater and inter-rater agreement by Kappa testing were 0.77 and 0.79 for TUBEX, 0.82 and 0.97 for Typhidot Rapid, and 0.87 and 0.95 for the Test-It assay.

**Table 2:** Characteristics of typhoid rapid diagnostic tests used in the study, Fiji, 2016

Characteristics	TUBEX^®^ TF, (IDL Biotec)	Typhidot Rapid (Reszon Diagnostic)	Test-It™ (Life Assay Diagnostics)
Assay and sample			
Target	*Salmonella typhi* IgM anti-09 antibodies	IgM antibodies to a 50 kDa *Salmonella typhi* outer membrane protein	IgM antibodies to *Salmonella enterica* LPS
Sample type	Serum (modified method for hemolyzed samples)	Serum/plasma/whole blood and finger-prick	Serum/plasma/whole blood and finger-prick
Sample volume	45 μL	30 μL serum/plasma, 35 μL whole blood	5 μL
Kit components (per box)			
Number of tests	36	25 individual cassettes	25 individual cassettes
Number of controls	Positive and negative control included in the kit	Not included (optional)	Not included
Consumables	No consumables included	No consumables included	All required consumables included
Reagents	Detector reagent, Indicator reagent. Wash buffer for hemolyzed samples optional.	Chase buffer	One bottle running fluid
Storage temperature (°C)			
Kit	2–28	4–30	4–28
Individual cassette	N/A	4–30	up to 45 for 2 months
Procedure			
Preparation time (per test run approx. 10 samples)	10–15 minutes	15 minutes	5–10 minutes
Incubation time (per test)	10 minutes	10 minutes (serum/plasma), 15–20 minutes (whole blood)	15 minutes
Result interpretation time (per test)	< 1 minute	< 1 minute	< 1 minute
Ease of use	Moderate	Easy	Easy
Result interpretation			
Method of interpretation	Visual color interpretation	The visual presence of a test line	The visual presence of a test line
Result score	Semiquantitative. 0 (negative) to 10 (strongly positive)	Qualitative only. A positive or negative result	Semiquantitative. Negative, 1+, 2+, 3+, 4+
Maximum incubation (time for interpretation)	30 minutes	Not stated	30 minutes
Ease to interpret	Difficult to classify based on color	Easy	Easy
Quality control			
Internal kit control	None	Individual cassette control line	Individual cassette control line and desiccant
Positive/negative controls	One positive and one negative control per run	Not included	Not included
Intra-rater Kappa	0.77 (0.67–0.86)	0.82 (0.73–0.91)	0.87 (0.78–0.97)
Inter-rater Kappa	0.79 (0.70–0.88)	0.97(0.94–1.00)	0.95 (0.89–1.00)
Cost			
Cost per test (USD)	3.1	2.2	2.0
Can be used in the field?	Yes. But more complicated than simple lateral flow RDTs	Yes. Simple lateral flow and can be used with finger-prick sample	Yes. Simple lateral flow and can be used with finger-prick sample

LPS = lipopolysaccharide; RDT = rapid diagnostic test.

**Figure 1.**
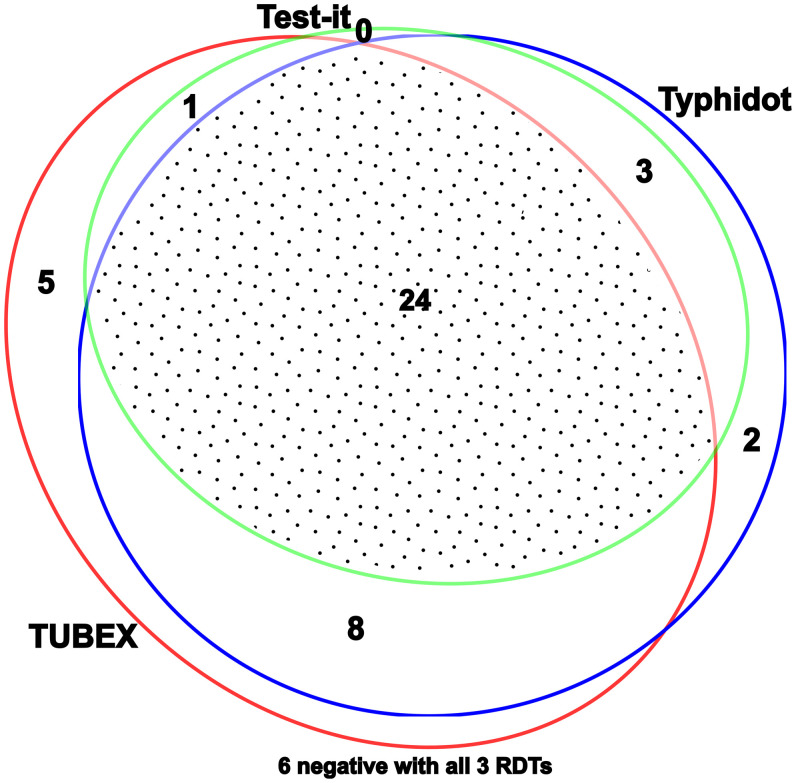
Positive results for each rapid diagnostic test (RDT) for the 49 patients with confirmed blood culture positive typhoid.

Of the total of 125 samples tested from participants with fever, 68 (54.4%) were positive by TUBEX, including 38 (77.6%) of 49 samples from typhoid cases and 30 (39.5%) of 76 samples from febrile controls, 48 (75.5%) were positive with Typhidot Rapid, including 37 (75.5%) of typhoid cases and 11 (14.5%) of febrile controls, and 33 (51.1%) positive with Test-It assay, including 28 (57.1%) of typhoid cases and 5 (16.6%) of febrile controls. The overlap of the RDT results in the confirmed positive cases is shown in Figure 1. There were 27 (21.6%) indeterminate results with the TUBEX, 9 (7.2%) with Typhidot Rapid, and 4 (3.2%) with the Test-It assay. In the febrile cases that were blood culture negative, there were two patients who were positive on two of the three RDTS and two that were positive with all three RDTs. These may have been misclassified patients with blood culture–negative typhoid.

For the community controls, nine (16.7%) tested positive with TUBEX and seven (13.0%) with Typhidot Rapid. The Test-It assay was negative in all community controls. The median (IQR) age of the community controls with a positive RDT (either TUBEX or Typhidot Rapid) was 35 (26–55) years compared with 25 (17–35) years for those with a negative RDT (*P* = 0.02). The median (IQR) anti-Vi IgG level of the community controls with a positive RDT (either TUBEX or Typhidot Rapid) was 65.4 (33.9–143.1) ELISA units compared with 39.0 (21.4–89.1) ELISA unit for those with a negative RDT (*P* = 0.33).

The sensitivity and specificity of the three RDTs using blood culture–confirmed typhoid cases as the reference standard are shown in Table 3. Table 3 also shows the PPV and NPVs of the different RDTs assuming a disease prevalence of 20%.^27^ TUBEX had a sensitivity of 77.6%, specificity 60.5%, with a PPV of 32.2%, and NPV of 91.5%. The sensitivity, specificity, PPV, and NPV of Typhidot Rapid were 75.5%, 85.5%, 56.6%, and 93.3%, respectively. The Test-It assay had a sensitivity of 57.1%, specificity 93.4%, PPV 68.5%, and NPV 89.7%.

**Table 3:** Sensitivity and specificity of the three rapid diagnostic tests for typhoid, Fiji, 2016

Rapid diagnostic tests	Typhoid cases Positive *N* = 49	Febrile controls Positive *N* = 76	Sensitivity % (95% CI)	Specificity % (95% CI)	Positive predictive value % (95% CI)	Negative predictive value % (95% CI)
TUBEX	38/49	30/76	77.6 (63.4–88.2)	60.5 (48.6–71.6)	32.9 (26.4–40.3)	91.5 (86.1–94.9)
Typhidot Rapid	37/49	11/76	75.5 (61.1–86.7)	85.5 (75.6–92.5)	56.6 (42.5–69.7)	93.3 (89.4–95.8)
Test-It Assay	28/49	5/76	57.1 (42.2–71.2)	93.4 (85.3–97.8)	68.5 (47.4–84.0)	89.7 (86.3–92.4)

The positive and negative predictive values were calculated assuming a disease prevalence of 20%.

We examined the use of binomial trials to attribute a febrile illness outbreak to typhoid. Supplemental Table 1 shows the binomial probabilities for one illustrative scenario where 15 febrile patients were tested, with a background typhoid prevalence of about 10%. The results indicate, for example, that in the absence of an outbreak, there is a probability of 0.002 (i.e., < 1/100) that six or more of these cases will have a positive test by chance alone. Therefore, a threshold of six or more positive cases could be used to raise a red flag that a typhoid outbreak is occurring, given it is very unlikely (< *P* = 0.01) that this would occur by chance.

The results of the simulation exercise are in Table 4 using the sensitivity and specificity estimates observed in our study. The low specificity of TUBEX resulted in a large number of false positives. For example, when there was only one TP case, on average six patients would test positive by the TUBEX test, resulting in an outbreak being declared in 68% of the 2,000 simulations even when there was no outbreak. In contrast, the Test-It method failed to detect many of the TP cases as a result of its low sensitivity. For example, when there were nine TP cases, the Test-It declared an outbreak in only 52% of simulations. The higher specificity of the Test-It method could potentially allow a smaller number of positives to be used as the threshold for declaring an outbreak. On balance, Typhidot had the best performance, with the median number testing positive being most similar to the correct number of positive cases. If there were one, six, and nine TP cases, the Typhidot declared an outbreak in 3.5%, 61%, and 92% of simulations (Table 4, column 5).

**Table 4:** Results of the simulation exercise for the results of the three typhoid RDTs performed on 15 subjects who form part of an outbreak of a febrile illness for attribution of the outbreak of febrile illness to typhoid

Number of true positive cases out of 15 (i.e., assumed to be correct value in the simulations)	Typhoid Rapid Diagnostic Test					
	TUBEX		Typhidot Rapid		Test-It	
	Number of positive results out of 15*	Proportion declared a typhoid outbreak†	Number of positive results out of 15*	Proportion declared a typhoid outbreak†	Number of positive results out of 15*	Proportion declared a typhoid outbreak†
1	6	0.678	3	0.035	1	0
2	7	0.753	3	0.068	2	0.006
3	7	0.803	4	0.145	2	0.012
4	7	0.861	5	0.263	3	0.031
5	8	0.897	5	0.418	3.5	0.070
6	8	0.934	6	0.614	4	0.150
7	9	0.967	6	0.754	4	0.249
8	9	0.972	7	0.853	5	0.383
9	9	0.985	8	0.919	6	0.523
10	10	0.989	8	0.964	6	0.632
11	10	0.995	9	0.977	7	0.729
12	11	0.998	9	0.990	7	0.802
13	11	0.998	10	0.995	8	0.876
14	11	1	11	0.998	8	0.905

RDT = rapid diagnostic test. The number of true positive required to declare an outbreak is set at 6 or more.

*Results shown are median numbers of tests declared positive, calculated over *N* = 2,000 simulated datasets each consisting of 15 patients.

†Proportion calculated over *N* = 2,000 simulations assuming an outbreak is declared if 6/15 tests are positive by the RDT.

## DISCUSSION

We assessed the diagnostic accuracy of the three RDTs for typhoid and examined their potential for use in the field setting for rapid outbreak identification in Fiji. Overall, the RDT’s performance was suboptimal, but could be used to correctly declare a typhoid outbreak if interpreted with care.

TUBEX had the highest sensitivity of 77.6%, but with a specificity of 60.5%, and PPV of 32.9%. The specificity of Test-It assay was the highest among all the tests at 93.4%. Moreover, the Test-It positive and NPVs were 68.5% and 89.7%, but its sensitivity was < 60%. Typhidot Rapid performed relatively well with specificity and sensitivity above 75% but PPV 56.6%. When tested on serum from community controls, TUBEX and Typhidot Rapid gave positive results in 16.7% and 13.0% of subjects, respectively, whereas Test-It assay gave no positive results. Community controls with a positive RDT result tended to be older, and with a higher level of anti-Vi IgG antibodies than those with a negative result, suggesting that prior typhoid may contribute to these positive results. The above findings suggest that none of the RDTs have sufficient accuracy to recommend them for individual patient diagnosis.

Our findings on the performance of Typhidot Rapid and Test-It assay are comparable to a recent systematic review, which reported sensitivity of 78% (95% CI 65–81) and 69 (95% CI 59–78) and specificity of 77 (95% CI 66–86) and 90 (95% CI 78–93), respectively.^20^ In our study, TUBEX sensitivity was similar to that found by the systematic review. TUBEX specificity was lower, at 60.5% (95% CI 49.5–71.5), in our study compared with the systematic review specificity of 87% (95% CI 82–91), and a study in Vietnam 94% (95% CI 71–100).^31^ A lower level of TUBEX specificity 69% (CI 95% 49.2–84.7) was also reported from a study in sub-Saharan African countries.^16^ Direct comparison of the diagnostic test performance could not be made as there are methodological differences between the studies as well as the level of typhoid endemicity. Repeated exposure or recent *S. typhi* infection could be one of the reasons for the lower specificity and high rate of false positivity of TUBEX in Fiji.

Outbreaks of typhoid in the community are frequent in Fiji.^4^^,^^21^^,^^32^^,^^33^ The number of outbreak-related typhoid cases varies from 15 to 30 patients. For example, after the Tropical Cyclone Winston, which hit Fiji on February 20, 2016, the MoHMS reported a total of 25 laboratory-confirmed cases and 26 suspected cases in Qelekuro and neighboring villages in the Central division, Tailevu subdivision, during 1-month period from March through April 2016. Blood culture is the only confirmatory test for typhoid in Fiji, and it is available in the three divisional-level hospitals. Therefore, a large outbreak of febrile illness of unknown etiology in an area remote to a blood culture service poses a major challenge for confirmation of the diagnosis. In this study, we have used a simulation exercise, using the binomial method, to identify a suitable alternative test for the rapid detection of an outbreak. We examined hypothetical situations of 15 febrile cases with variable true prevalence of typhoid. Our simulations demonstrated that Typhidot Rapid test performed better than the other RDTs in the outbreak simulation scenario declaring an outbreak in 61% of simulations when the threshold of six TP cases had been reached. TUBEX overcalled outbreaks and Test-It assay undercalled them. If the threshold for declaring an outbreak was lower than six TP, the higher specificity of the Test-It method might also allow it to be used. The results suggest that the Typhoidot Rapid test and the Test-It method may have a potential use during a suspected typhoid outbreak in the investigation in the community. We could have proposed a more liberal threshold for declaring an outbreak (e.g., 5/15), but run the risk of declaring more frequent false positive outbreaks. The detection of potential outbreaks might be followed up by more definitive blood culture testing in a two-step process.

Our laboratory staff found that the TUBEX test was technically more challenging to perform than the Typhidot Rapid and Test-It assays. The intra-rater and inter-rater agreement were slightly less for TUBEX, compared with the Typhidot Rapid and Test-It assays, and the cost is higher. The intra- and inter-Kappa for both tests were > 80% indicating very good reproducibility of results by the individual observer as well as with that of other observers. Test-It assay included all required consumables making it the most convenient and cheapest of all the three tests we evaluated.

Our study has limitations. We used blood culture as a gold standard for typhoid diagnosis, despite its limited sensitivity. Some typhoid patients may have been misclassified as non-cases by blood culture, incorrectly classifying some RDT-positive participants false positive. Four of the febrile controls with a negative blood culture were positive by two or three of the RDTs and may represent such misclassifications. Another limitation is the design with previously collected cases and controls, rather than consecutive patients presenting with fever, and the small number of patients included in the study. The ELISA tests used for leptospirosis and dengue do not have perfect sensitivity and specificity, which may have resulted in misclassification of febrile controls.

In conclusion, the performance of the typhoid RDTs was generally suboptimal with low sensitivity (less than 80%) and variable specificity (60–93%). Although we do not recommend their use in Fiji for patient-level diagnosis of typhoid, they may be useful in the field investigation of outbreaks of febrile illness for rapid assessment of typhoid as a cause. Of the three assays evaluated, Typhidot Rapid had the best combination of sensitivity, specificity, PPV, NPV, cost, and ease of use for this purpose, but further evaluations of its use in the field are required.

## Supplemental Material


Supplemental materials
