# Supplementary material for: Assessment of Rapid Diagnostic Tests for Typhoid Diagnosis and Assessment of Febrile Illness Outbreaks in Fiji
Source: Am J Trop Med Hyg. 2021 Nov 29;106(2):543–9. doi: 10.4269/ajtmh.21-0771 (PMC8832939; doi:10.4269/ajtmh.21-0771)
Supplement: Supplementary file 1 [file tpmd210771.SD1.pdf]

Supplementary table 1 Binomial probabilities of a positive typhoid test assuming a background typhoid positivity rate of 10% among febrile cases, n=15 febrile cases

Number of positives (X)	Probability (exactly X positives)	Cumulative probability (>=X positives)
0	0.21	1.00
1	0.34	0.79
2	0.27	0.45
3	0.13	0.18
4	0.04	0.06
5	0.01	0.01
6	0.002	0.002
7	0.0003	0.0003
8	0.00003	0.00003
9	<0.00001	<0.00001
10	<0.00001	<0.00001
11	<0.00001	<0.00001
12	<0.00001	<0.00001
13	<0.00001	<0.00001
14	<0.00001	<0.00001
15	<0.00001	<0.00001
